# International Differences in Multiple Sclerosis Health Outcomes and Associated Factors in a Cross-sectional Survey

**DOI:** 10.3389/fneur.2017.00229

**Published:** 2017-05-31

**Authors:** Grace D. Reilly, Awng Shar Mahkawnghta, Pia L. Jelinek, Alysha M. De Livera, Tracey J. Weiland, Chelsea R. Brown, Keryn L. Taylor, Sandra L. Neate, George A. Jelinek, Claudia H. Marck

**Affiliations:** ^1^Melbourne School of Population and Global Health, University of Melbourne, Melbourne, VIC, Australia; ^2^Sir Charles Gairdner Hospital, Nedlands, WA, Australia; ^3^Neuroepidemiology Unit, Melbourne School of Population and Global Health, University of Melbourne, Melbourne, VIC, Australia; ^4^Biostatistics Unit, Melbourne School of Population and Global Health, University of Melbourne, Melbourne, VIC, Australia

**Keywords:** multiple sclerosis, disability, quality of life, fatigue, depression

## Abstract

Multiple sclerosis (MS) is a major cause of disability and poor quality of life (QOL). Previous studies have shown differences in MS health outcomes between countries. This study aimed to examine the associations between international regions and health outcomes in people with MS. Self-reported data were taken from the Health Outcomes and Lifestyle In a Sample of people with Multiple Sclerosis online survey collected in 2012. The 2,401 participants from 37 countries were categorized into three regions: Australasia, Europe, and North America. Differences were observed between regions in disability, physical and mental health QOL, fatigue, and depression, but most of these disappeared after adjusting for sociodemographic, disease, and lifestyle factors in multivariable regression models. However, adjusted odds for disability were higher in Europe [odds ratio (OR): 2.17, 95% confidence interval (CI): 1.28 to 3.67] and North America (OR: 1.79, 95% CI: 1.28 to 2.51) compared to Australasia. There may be other unmeasured factors that vary between regions, including differences in access and quality of healthcare services, determining disability in MS. When assessing differences in MS health outcomes, lifestyle factors and medication use should be taken into consideration.

## Introduction

Multiple sclerosis (MS) is a chronic autoimmune neurological disorder involving inflammation and demyelination of the central nervous system (CNS). An estimated 2.5 million people are affected by MS across the world. MS can cause debilitating physical and mental health symptoms and often affects young people. Symptoms vary and depend on which part of the CNS is affected and include numbness, tingling, fatigue, and problems with balance and vision. Common comorbidities of MS are back pain, arthritis, anxiety, and depression (1). There is a genetic predisposition to MS, however, genetics play little role in progression, severity and presence of comorbidities, which have been closely linked to environmental and lifestyle factors (1–3). There is a higher prevalence of MS in developed countries compared to developing countries. Furthermore, prevalence increases as latitude from the equator increases thought to be associated with sun exposure and vitamin D (4). Other environmental risk factors for MS onset and progression include infections, and lifestyle factors such as smoking, stress, diet, and weight (5–7).

Multiple sclerosis is one of the major causes of disability in young adults in developed countries and is associated with poorer quality of life (QOL) than the general population (8, 9). Fatigue is the most common symptom affecting people with MS (PwMS) with a prevalence of up to 75%, significantly higher than the general population (10, 11). Depression is the most common mental health comorbidity of MS with a lifetime prevalence of up to 50%, which is markedly elevated compared to the general population (10–15%), and higher compared to people with other chronic illnesses (12–14). Comparisons of these health outcomes for different countries or regions are difficult to interpret because the tools used to measure health outcomes and inclusion and exclusion criteria vary between studies. To date, few studies have compared health outcomes such as disability, physical and mental health QOL, fatigue, and depression in PwMS between countries globally.

We have previously reported international differences in obesity and many comorbidities including depression, diabetes, and cardiovascular disease in PwMS, with a higher prevalence in the US and Canada using date from the Health Outcomes and Lifestyle In a Sample of people with Multiple Sclerosis (HOLISM) study (14). Lifestyle factors, including diet, smoking, and physical activity, were also associated with a number of comorbidities (14). Differences in other health outcomes are also likely to be associated with differences in health care access, latitude, lifestyle, and other sociodemographic factors that vary from country to country (15, 16).

The aim of the current study was to assess differences in health outcomes between countries and assess if these differences were associated with sociodemographic, disease, and lifestyle variables in the HOLISM study.

## Materials and Methods

The methodology of the HOLISM study has previously been described in detail (17). A summary of the relevant details is provided here.

### Participants and Procedures

Participants were recruited via MS society websites and newsletters, MS blogs, MS forums, Facebook pages, and Twitter accounts specific for PwMS over 15 weeks in 2012. The study ad and link to the survey were posted through these media, many of which had a health and lifestyle focus and were in English language. The web-based tool, SurveyMonkey^®^, was used to provide respondents with an information sheet, electronic consent indicator, and the survey itself. To be eligible for the study, participants were required to be over 18 years of age and to have a self-reported physician-confirmed diagnosis of MS. Contact details from all participants were required to facilitate follow-up. The study was approved by the Health Sciences Human Ethics Sub-Committee at the University of Melbourne (Ethics ID: 1545102), and all participants indicated consent before entering the survey.

### Measures

The online survey was in English and used validated tools where possible. It took approximately 40 min to complete, and all data were self-reported.

#### Sociodemographic and Disease Characteristics

The sociodemographics and disease characteristics that were assessed included age, gender, years since diagnosis, age at diagnosis, type of MS, level of disability, disease-modifying drug (DMD) use, employment status, marital status, education level, and latitude derived from data for location (country and city). Country of residence was grouped into three categories that had sufficient sample size to enable meaningful analysis: Australasia (Australia and New Zealand), Europe, and North America (United States and Canada). Participants from Africa, Asia, and Latin America were excluded because of small sample size.

#### Disability

Current level of disability was measured using a self-reported Patient Determined Disease Step (PDDS) score. The PDDS has been validated against the EDDS, which is commonly used by neurologists and clinicians (18). PDDS was categorized into a binary outcome, such that scores of 0–3 were classified as able to walk unassisted or “no disability” and 4–8 as unable to walk unassisted or “disability.”

#### Quality of Life

The Multiple Sclerosis Quality Of Life-54 scale was used to measure health-related QOL and was specifically designed for PwMS (19). Scores range from zero to 100, indicating lowest to highest QOL, respectively. The physical composite score accounts for factors such as physical role limitation, energy/fatigue, pain, and sexual function. The mental health composite includes health distress, emotional wellbeing, emotional role limitation, and cognitive function (19).

#### Fatigue

The Fatigue Severity Scale (20) is a validated tool that consists of nine fatigue-related statements that are rated over the past week on a seven-point scale from “disagree” to “agree.” A mean score of ≥4 was used as a cutoff to indicate clinically significant fatigue as is commonly done.

#### Depression

Depression risk was assessed using the two-item Patient Health Questionnaire-2, which has been validated for use in PwMS (21). A cutoff of ≥3 is commonly used as a positive screen for depression risk.

#### Modifiable Lifestyle Factors

A range of lifestyle factors was assessed. Dietary habits were assessed using a modified version of the Diet Habits Questionnaire in which items assessing salt intake and alcohol use were excluded. A higher score meant a better diet as prescribed for cardiac health (22). Alcohol consumption was instead assessed in more detail separately and was classified as low (<15 g/week) and moderate/high consumption (≥15 g/week). Physical activity level was assessed using the International Physical Activity Questionnaire (23). Social support was measured using the Single Item Measure of Social Support (24). Other lifestyle factors assessed with researcher-devised questions were smoking status, vitamin D supplementation, omega-3 supplementation, type of omega-3 supplementation, meditation frequency, and body mass index (BMI, derived from weight and height). BMI was classified as underweight (<18.5 kg/m^2^), normal (18.5–24.9 kg/m^2^), overweight (25.0–29.9 kg/m^2^), and obese (≥30 kg/m^2^).

### Data Analysis

Stata 13.0 was used to analyze the data of this cross-sectional study. Sample characteristics were first described to examine sociodemographics, disease characteristics, and modifiable lifestyle factors of participants overall and within each region (Australasia, Europe, and North America). Categorical, continuous, and discrete or skewed data were presented as frequency (percentages), mean (standard deviation), and median (25th–75th percentile), respectively. Multivariable linear and logistic regression models were used to explore the associations between regions and the health outcomes, adjusting for confounders identified *a priori* based on our previous research (11, 13, 25, 26). We assessed for any potential interactions between region and sociodemographics, disease characteristics, and modifiable lifestyle factors using likelihood ratio tests. No significant interactions were found for any of the health outcomes and were therefore not included in the models.

## Results

Descriptive data outlining regional differences in sociodemographics, disease characteristics, and lifestyle factors are presented in Table 1. Significant differences between the regions existed in nearly all variables.

**Table 1:** Characteristics of the study sample by region.

	*N* (%) unless otherwise specified	Australasia	Europe	North America	Total
Gender*	Male	145/789 (18.4)	129/601 (21.5)	114/848 (13.4)	388/2,238 (17.3)
	Female	644/789 (81.6)	472/601 (78.5)	734/848 (85.6)	1,850/2,238 (82.7)

Age* (years)	Mean (SD)	47.1 (10.8)	43.7 (10.5)	45.8 (10.0)	45.7 (10.5)

Marital status*	Married/cohabiting/partnered	636/826 (77.0)	445/638 (69.8)	668/900 (74.2)	1,749/2,364 (74.0)
	Single	112/826 (13.6)	123/638 (19.3)	102/900 (11.3)	337/2,364 (14.3)
	Separated/divorced/widower	78/826 (9.4)	70/638 (11.0)	130/900 (14.4)	278/2,364 (11.8)

Employment status*	Employed full or part time	482/818 (58.9)	364/626 (58.2)	437/895 (48.8)	1,283/2,339 (54.9)
	Student or stay at home carer	100/818 (12.2)	55/626 (8.8)	84/895 (9.4)	239/2,339 (10.2)
	Unemployed	56/818 (6.9)	61/626 (9.7)	69/895 (7.7)	186/2,339 (8.0)
	Retired due to age or disability	180/818 (22.0)	146/626 (23.3)	305/895 (34.1)	631/2,339 (27.0)

Education level*	No school/primary/secondary	233/833 (28.0)	128/645 (19.8)	231/909 (25.4)	592/2,387 (24.8)
	Vocational training	147/833 (17.7)	93/645 (14.4)	147/909 (16.2)	387/2,387 (16.2)
	Bachelor degree	309/833 (37.1)	236/645 (36.6)	316/909 (34.8)	861/2,387 (36.1)
	Postgraduate	144/833 (17.3)	188/645 (29.2)	215/909 (23.7)	547/2,387 (22.9)

**Disease characteristics**					

Years since diagnosis (quartiles)	Less than 3 years	225/820 (27.4)	191/637 (30.0)	269/896 (30.0)	685/2,353 (29.1)
	4–6 years	183/820 (22.3)	140/637 (22.0)	201/896 (22.4)	524/2,353 (22.3)
	7–12 years	210/820 (25.6)	155/637 (24.3)	209/896 (23.3)	574/2,353 (24.4)
	13 years and above	202/820 (24.6)	151/637 (23.7)	217/896 (24.2)	570/2,353 (24.2)

Age at diagnosis*	Mean (SD)	39.2 (10.2)	36.2 (9.8)	38.1 (10.1)	38.0 (10.1)

Type of MS*	Benign	43/822 (5.2)	29/639 (4.6)	22/894 (2.5)	94/2,355 (4.0)
	Relapsing remitting	500/822 (60.8)	379/639 (59.3)	577/894 (64.5)	1,456/2,355 (61.8)
	Primary progressive	79/822 (9.6)	39/639 (6.1)	55/894 (6.2)	173/2,355 (7.4)
	Secondary progressive	67/822 (8.2)	83/639 (13.0)	119/894 (13.3)	269/2,355 (11.4)
	Progressive relapsing	14/822 (1.7)	13/639 (2.0)	19/894 (2.1)	46/2,355 (2.0)
	Unsure/other	119/822 (14.5)	96/639 (15.0)	102/894 (11.4)	319/2,355 (13.5)

Current disease modifying medication use*	No current DMD use	394/778 (50.6)	365/590 (61.9)	351/803 (43.7)	1,110/2,171 (51.1)
	Less than 12 months	120/778 (15.4)	71/590 (12.0)	137/803 (17.1)	328/2,171 (15.1)
	More than 12 months	264/778 (33.9)	154/590 (26.1)	315/803 (39.2)	733/2,171 (33.8)

**Modifiable lifestyle factors**					

Smoking status*	Never	397/790 (50.3)	286/601 (47.6)	383/835 (45.9)	1,066/2,226 (47.9)
	Former	327/790 (41.4)	241/601 (40.1)	335/835 (40.1)	903/2,226 (40.6)
	Current	66/790 (8.4)	74/601 (12.3)	117/835 (14.0)	257/2,226 (11.6)

Alcohol consumption*	Low	439/785 (55.9)	343/598 (57.4)	573/828 (69.2)	1,355/2,211 (61.3)
	Moderate and high (>15 g/week)	346/785 (44.1)	255/598 (42.6)	255/828 (30.8)	856/2,211 (38.7)

Physical activity level*	Low	305/780 (39.1)	242/587 (41.2)	338/803 (42.1)	885/2,170 (40.8)
	Moderate	253/780 (32.4)	213/587 (36.3)	225/803 (28.0)	691/2,170 (31.8)
	High	222/780 (28.5)	132/587 (22.5)	240/803 (29.9)	594/2,170 (27.4)

Vitamin D supplementation*	None	95/755 (12.6)	140/574 (24.4)	138/805 (17.1)	373/2,134 (17.5)
	1–5,000 IU	485/755 (64.2)	344/574 (59.9)	492/805 (61.1)	1,321/2,134 (61.9)
	>5,000 IU	175/755 (23.2)	90/574 (15.7)	175/805 (21.7)	440/2,134 (20.6)

Omega 3 supplementation type*	None	220/773 (28.5)	214/576 (37.2)	345/789 (43.7)	779/2,138 (36.4)
	Fish oil	270/773 (34.9)	206/576 (35.8)	301/789 (38.2)	777/2,138 (36.3)
	Flaxseed oil	96/773 (12.4)	63/576 (10.9)	38/789 (4.8)	197/2,138 (9.2)
	Both fish oil and flaxseed oil	187/773 (24.2)	93/576 (16.2)	105/789 (13.3)	385/2,138 (18.0)

Fish consumption*	Less than once per week	131/794 (16.5)	130/599 (21.7)	373/833 (44.8)	634/2,226 (28.5)
	1–2 times per week	325/794 (40.9)	233/599 (38.9)	341/833 (40.9)	899/2,226 (40.4)
	3 or more days per week	338/794 (42.6)	236/599 (39.4)	119/833 (14.3)	693/2,226 (31.1)

Meditation frequency*	Never	293/783 (37.4)	328/591 (55.5)	412/807 (51.1)	1,033/2,181 (47.4)
	Less than once per week	214/783 (27.3)	119/591 (20.1)	159/807 (19.7)	492/2,181 (22.6)
	1–4 times per week	194/783 (24.8)	100/591 (16.9)	139/807 (17.2)	433/2,181 (19.9)
	5 or more times per week	82/783 (10.5)	44/592 (7.5)	97/807 (12.0)	223/2,181 (10.2)

Social Support*	0 or 1 support person	204/781 (26.1)	175/583 (30.0)	204/801 (25.5)	583/2,165 (26.9)
	2 or more support people	577/781 (73.9)	408/583 (70.0)	597/801 (74.5)	1,582/2,165 (73.1)

Body Mass Index (kg/m^2^)*	Normal	600/822 (73.0)	475/636 (74.7)	647/900 (71.9)	1,722/2,358 (73.0)
	Underweight	35/822 (4.3)	31/636 (4.9)	37/900 (4.1)	103/2,358 (4.4)
	Overweight	187/822 (22.7)	130/636 (20.4)	216/900 (24.0)	533/2,358 (22.6)
	Obese	130/822 (15.8)	78/636 (12.3)	249/900 (27.7)	457/2,358 (19.4)

Dietary habits score*	Mean (SD)	86.0 (76.5–92.0)	81.0 (72.0–89.5)	75.0 (66.7–83.0)	80.5 (70.4–89.0)
Latitude*	Mean (SD)	35.5 (4.7)	51.7 (4.5)	39.6 (5.7)	41.5 (8.3)

**Median (25th–75th percentile).

***^#^**The p-value obtained from a Chi-square test assessing whether there is a relationship between the variable and the regions is less than 0.05*.

Descriptive data on major health outcomes (disability, physical and mental health QOL, clinically significant fatigue, and depression risk) in our sample are presented per region in Table 2. Significant differences existed between regions for all health outcomes.

**Table 2:** Health outcomes by region.

		Number (%) unless otherwise stated			
		Australasia	Europe	North America	Total
Disability^a^	Able to walk unassisted	603/796 (75.7)	417/603 (69.2)	566/837 (67.6)	1,586/2,236 (70.9)
	Unable to walk unassisted	193/796 (24.3)	186/603 (30.8)	271/837 (32.4)	650/2,236 (29.1)
Clinically significant fatigue^a^	Not fatigued	282/750 (37.6)	196/562 (34.9)	237/767 (30.9)	715/2,079 (34.4)
	Fatigued	468/750 (62.4)	366/562 (65.1)	530/767 (69.1)	1,364/2,079 (65.6)
Depression risk^a^	Negative depression screen	660/773 (85.4)	477/589 (81.0)	620/804 (77.1)	1,757/2,166 (81.1)
	Positive depression screen	113/773 (14.6)	112/589 (19.0)	184/804 (22.9)	409/2,166 (18.9)
Quality of life	Physical^a^^,^^b^	63.7 (20.3)	58.1 (21.5)	55.8 (21.8)	59.1 (21.5)
	Mental^a^^,^^b^	70.8 (19.8)	65.8 (21.2)	63.7 (22.3)	66.8 (21.4)

*^a^The p-value obtained from a Chi-square test assessing whether there is a relationship between the variable and the regions is less than 0.05*.

*^b^Mean (SD)*.

Multivariable regression models for disability, physical QOL, mental health QOL, clinically significant fatigue, and depression risk were adjusted for sociodemographic, disease, and lifestyle determinants (Tables 3 and 4). Adjusted odds for disability were higher in Europe and North America compared to Australasia (Table 4). Adjusted odds for physical and mental health QOL, clinically significant fatigue, and depression risk were not significantly associated with region (Tables 3 and 4).

**Table 3:** Associations between regions and other covariates with physical and mental health quality of life (QOL) obtained using multivariable linear regression models.

Variables		Physical QOL (*n* = 1,602)^a^		Mental health QOL (*n* = 1,766)^b^	
		Mean differences (95% CI)	*p*-Value	Mean differences (95% CI)	*p*-Value
Region	Australasia	Reference	–	Reference	–
	Europe	−2.88 (−6.41, 0.65)	0.110	0.27 (−3.52, 4.06)	0.888
	North America	−1.24 (−3.46, 0.98)	0.273	−0.96 (−3.36, 1.43)	0.431

**Sociodemographic factors**					

Gender	Male	Reference	–	Reference	–
	Female	0.67 (−1.54, 2.89)	0.550	1.37 (−1.09, 3.83)	0.276
	Age (10 years)	−1.37 (−2.36, −0.38)	0.007	3.05 (1.94, 4.16)	<0.001

Marital status	Married/cohabiting/partnered	–	–	Reference	–
	Single	–	–	−2.81 (−5.62, −0.01)	0.049
	Separated/divorced/widower	–	–	−3.26 (−6.27, −0.24)	0.034

Employment status	Employed full or part time	Reference	–	Reference	–
	Student or stay at home carer	−1.00 (−3.77, 1.77)	0.478	−1.43 (−4.48, 1.61)	0.356
	Unemployed	−6.29 (−9.69, −2.88)	<0.001	−9.90 (−13.54, −6.26)	<0.001
	Retired due to age or disability	−13.20 (−15.49, −10.92)	<0.001	−7.11 (−9.61, −4.62)	<0.001

Education level	No school/primary/secondary	Reference	–	Reference	–
	Vocational training	0.89 (−1.76, 3.55)	0.510	1.30 (−1.57, 4.17)	0.374
	Bachelor degree	3.66 (1.40, 5.93)	0.002	1.73 (−0.71, 4.17)	0.164
	Postgraduate	3.08 (0.57, 5.59)	0.016	2.20 (−0.52, 4.92)	0.113

**Disease characteristics**					

Years since diagnosis (quartiles)	Less than 3 years	Reference	–	Reference	–
	4–6 years	0.35 (−2.05, 2.75)	0.775	−0.37 (−2.98, 2.24)	0.781
	7–12 years	−2.96 (−5.36, −0.56)	0.016	−1.14 (−3.76, 1.48)	0.392
	13 years and above	−2.82 (−5.40, −0.23)	0.033	−1.38 (−4.24, 1.47)	0.343

Type of MS	Relapsing-remitting	–	–	Reference	–
	Primary progressive	–	–	−2.28 (−6.11, 1.54)	0.242
	Secondary progressive	–	–	−2.99 (−6.23, 0.25)	0.070
	Progressive relapsing	–	–	−5.50 (−12.24, 1.24)	0.109
	Benign	–	–	5.37 (0.59, 10.16)	0.028
	Unsure/other	–	–	−1.88 (−4.69, 0.94)	0.191

Current disease modifying medication use	No current DMD use	Reference	–	Reference	–
	Less than 12 months	−2.53 (−5.07, 0.02)	0.052	−3.24 (−6.03, −0.44)	0.023
	More than 12 months	−1.14 (−3.03, 0.76)	0.240	−1.96 (−4.11, 0.20)	0.075

**Modifiable lifestyle factors**					

Smoking status	Never	Reference	–	Reference	–
	Former	−0.87 (−2.66, 0.92)	0.339	−2.30 (−4.22, −0.37)	0.020
	Current	−7.77 (−10.61, −4.93)	<0.001	−7.14 (−10.27, −4.02)	<0.001

Alcohol consumption	Low	Reference	–	Reference	–
	Moderate and high	3.66 (1.91, 5.41)	<0.001	2.42 (0.52, 4.32)	0.013

Physical activity level	Low	Reference	–	Reference	–
	Moderate	9.39 (7.37, 11.42)	<0.001	4.53 (2.33, 6.72)	<0.001
	High	15.05 (12.93, 17.18)	<0.001	6.97 (4.63, 9.30)	<0.001

Vitamin D supplementation	None	Reference	–	Reference	–
	1–5,000 IU	−0.45 (−2.87, 1.97)	0.714	0.99 (−1.63, 3.61)	0.458
	>5,000 IU	0.64 (−2.26, 3.55)	0.665	1.77 (−1.39, 4.92)	0.272

Omega 3 supplementation type	None	Reference	–	Reference	–
	Fish oil	1.79 (−0.32, 3.89)	0.096	0.62 (−1.66, 2.89)	0.594
	Flaxseed oil	3.10 (−0.18, 6.37)	0.064	1.44 (−2.09, 4.97)	0.424
	Both fish oil and flaxseed oil	3.04 (0.33, 5.75)	0.028	−0.29 (−3.19, 2.60)	0.842

Meditation frequency	Never	Reference	–	Reference	–
	Less than once per week	−1.36 (−3.48, 0.77)	0.212	−2.59 (−4.88, −0.30)	0.027
	1–4 times per week	−2.23 (−4.62, 0.16)	0.068	0.21 (−2.37, 2.79)	0.874
	5 or more times per week	−1.52 (−4.46, 1.43)	0.313	2.56 (−0.66, 5.77)	0.119

Social support	0 or 1 support person	Reference	–	Reference	–
	2 or more support people	4.42 (2.52, 6.32)	<0.001	7.15 (5.10, 9.19)	<0.001

Body mass index (kg/m^2^)	Normal	Reference	–	Reference	–
	Underweight	2.22 (−2.05, 6.49)	0.309	2.38 (−2.11, 6.87)	0.298
	Overweight	−3.03 (−5.14, −0.91)	0.005	−3.24 (−5.54, −0.94)	0.006
	Obese	−5.74 (−8.08, −3.40)	<0.001	−4.50 (−7.03, −1.98)	<0.001

Dietary habits score (10 U)		2.05 (1.16, 2.94)	<0.001	2.23 (1.27, 3.20)	<0.001

Latitude (10°)		−0.69 (−2.33, 0.95)	0.407	−1.24 (−3.03, 0.56)	0.176

*Complete case analysis was used*.

*^a^Analyses consisted of 66.7% participants. Marital status was not included as a covariate in the model as it is not directly associated with physical quality of life (26), and type of MS was not considered to be confounders in this model*.

*^b^Analyses consisted of 73.6% participants*.

**Table 4:** Associations between regions and other covariates with clinically significant fatigue, depression screen, disability obtained using multivariable logistic regression.

Variables		Clinically significant fatigue (n = 1,735)^a^		Depression risk (*n* = 1,779)^b^		Disability (*n* = 1,862)^c^	
		AOR (95% CI)	*p*-Value	AOR (95% CI)	*p*-Value	AOR (95% CI)	*p*-Value
Region	Australasia	Reference	–	Reference	–	Reference	–
	Europe	1.12 (0.69, 1.80)	0.650	1.02 (0.59, 1.78)	0.932	2.17 (1.28, 3.67)	0.004
	North America	0.87 (0.64, 1.18)	0.366	1.13 (0.79, 1.61)	0.507	1.79 (1.28, 2.51)	0.001

**Sociodemographic factors**							

Gender	Male	Reference	–	Reference	–	Reference	–
	Female	1.21 (0.90, 1.64)	0.210	0.74 (0.51, 1.07)	0.11	0.64 (0.46, 0.89)	0.009

Age (10 years)		1.02 (0.89, 1.17)	0.749	0.77 (0.65, 0.91)	0.002	2.08 (1.80, 2.39)	<0.001

Marital status	Married/cohabiting/partnered			Reference	–		
	Single			1.34 (0.90, 1.99)	0.148		
	Separated/divorced/widower			1.33 (0.89, 2.01)	0.169		

Employment status	Employed full or part time	Reference	–	Reference	–		
	Student or stay at home carer	1.16 (0.80, 1.67)	0.439	0.75 (0.45, 1.23)	0.251		
	Unemployed	1.25 (0.80, 1.96)	0.324	1.78 (1.09, 2.91)	0.021		
	Retired due to age or disability	2.33 (1.66, 3.27)	<0.001	1.42 (1.00, 2.02)	0.053		

Education status	No school/primary/secondary	Reference	–	Reference	–	Reference	–
	Vocational training	0.71 (0.48, 1.04)	0.077	1.02 (0.69, 1.52)	0.902	1.04 (0.72, 1.51)	0.831
	Bachelor degree	0.65 (0.47, 0.89)	0.007	0.85 (0.60, 1.21)	0.371	0.80 (0.58, 1.11)	0.185
	Postgraduate	0.53 (0.37, 0.75)	<0.001	0.74 (0.49, 1.11)	0.148	0.62 (0.43, 0.90)	0.013

**Disease characteristics**							

Years since diagnosis (quartiles)	Less than 3 years	Reference	–	Reference	–	Reference	–
	4–6 years	0.84 (0.61, 1.15)	0.273	1.33 (0.88, 2.01)	0.17	2.56 (1.68, 3.90)	<0.001
	7–12 years	1.20 (0.87, 1.67)	0.268	1.68 (1.12, 2.50)	0.012	4.41 (2.95, 6.59)	<0.001
	13 years and above	1.00 (0.70, 1.43)	0.998	2.01 (1.29, 3.12)	0.002	5.34 (3.55, 8.04)	<0.001

Type of MS	Relapsing-remitting	Reference	–	Reference	–		
	Primary progressive	1.90 (1.11, 3.25)	0.019	1.33 (0.79, 2.24)	0.289		
	Secondary progressive	1.44 (0.92, 2.23)	0.107	0.99 (0.63, 1.57)	0.981		
	Progressive relapsing	3.12 (0.86, 11.34)	0.084	1.39 (0.60, 3.24)	0.44		
	Benign	0.40 (0.23, 0.71)	0.002	0.56 (0.21, 1.48)	0.241		
	Unsure/other	1.02 (0.71, 1.44)	0.933	0.93 (0.60, 1.43)	0.731		

Current disease modifying medication use	No current DMD use	Reference	–	Reference	–	Reference	–
	Less than 12 months	1.34 (0.95, 1.89)	0.097	1.46 (0.96, 2.22)	0.077	1.05 (0.69, 1.60)	0.81
	More than 12 months	1.48 (1.13, 1.94)	0.05	1.41 (1.03, 1.94)	0.034	0.71 (0.54, 0.95)	0.02

**Modifiable lifestyle factors**							

Smoking status	Never	Reference	–	Reference	–	Reference	–
	Former	0.86 (0.68, 1.09)	0.210	1.34 (1.00, 1.79)	0.053	1.26 (0.96, 1.64)	0.091
	Current	1.54 (0.99, 2.39)	0.057	2.22 (1.48, 3.31)	<0.001	1.57 (1.04, 2.37)	0.033

Alcohol	Low	Reference	–	Reference	–	Reference	
	Moderate and high	0.68 (0.54, 0.86)	0.001	0.71 (0.53, 0.95)	0.022	0.76 (0.58, 0.99)	0.042

Physical activity level	Low	Reference	–	Reference	–	Reference	–
	Moderate	0.50 (0.38, 0.67)	<0.001	0.60 (0.43, 0.82)	0.002	0.23 (0.17, 0.31)	<0.001
	High	0.29 (0.22, 0.39)	<0.001	0.40 (0.28, 0.58)	<0.001	0.18 (0.13, 0.25)	<0.001

Vitamin D supplementation	None	Reference	–	Reference	–	Reference	–
	1–5,000 IU	0.70 (0.49, 1.00)	0.047	0.88 (0.62, 1.25)	0.476	1.20 (0.85, 1.70)	0.306
	>5,000 IU	0.62 (0.41, 0.94)	0.025	0.86 (0.55, 1.36)	0.523	1.33 (0.86, 2.06)	0.198

Omega 3 supplementation type	None	Reference	–	Reference	–	Reference	–
	Fish oil	0.94 (0.71, 1.26)	0.681	0.76 (0.55, 1.04)	0.089	0.83 (0.61, 1.13)	0.247
	Flaxseed oil	0.75 (0.49, 1.16)	0.196	0.62 (0.34, 1.11)	0.107	0.78 (0.48, 1.27)	0.315
	Both fish oil and flaxseed oil	1.25 (0.87, 1.81)	0.226	0.74 (0.47, 1.15)	0.181	0.71 (0.48, 1.07)	0.105

Meditation frequency	Never	Reference	–	Reference	–		
	Less than once per week	1.40 (1.05, 1.87)	0.022	1.25 (0.89, 1.74)	0.194		
	1–4 times per week	1.29 (0.94, 1.78)	0.114	1.03 (0.69, 1.56)	0.872		
	5 or more times per week	1.64 (1.07, 2.50)	0.022	0.62 (0.36, 1.07)	0.083		

Social support	0 or 1 support person	Reference	–	Reference	–	Reference	–
	2 or more support people	0.77 (0.59, 1.00)	0.052	0.45 (0.34, 0.59)	<0.001	1.02 (0.78, 1.34)	0.886

Body mass index (kg/m^2^)	Normal	Reference	–	Reference	–	Reference	–
	Underweight	0.82 (0.47, 1.41)	0.466	0.86 (0.43, 1.74)	0.678	1.16 (0.62, 2.17)	0.638
	Overweight	1.38 (1.03, 1.83)	0.029	1.15 (0.82, 1.62)	0.405	1.02 (0.75, 1.40)	0.896
	Obese	1.93 (1.37, 2.73)	<0.001	1.36 (0.96, 1.94)	0.083	1.45 (1.04, 2.02)	0.029

Dietary habits score (10 U)		0.81 (0.72, 0.92)	0.001	0.84 (0.73, 0.96)	0.012	1.04 (0.92, 1.19)	0.504
Latitude (10°)		1.06 (0.84, 1.33)	0.619	1.06 (0.82, 1.38)	0.652	0.99 (0.77, 1.27)	0.914

*^a^Complete case analysis was used. Analyses consisted of 72.3% of the participants. Marital status was not included as a covariate as it was not directly associated with fatigue*.

*^b^Complete case analysis was used. Analyses consisted of 74.1% of the participants*.

*^c^Complete case analysis was used. Analyses consisted of 77.6% of the participants. Marital status was not included as a covariate as it was not directly associated with disability. Type of MS and meditation were not included due to reverse causality (25)*.

Many of the sociodemographic and disease characteristics and modifiable lifestyle factors had independent associations with the health outcomes. The main variables associated with a clinically significant difference (5 points) in physical and mental QOL score were age, employment status, type of MS, smoking status, physical activity, social support, BMI, and diet (Table 3).

The main variables associated with a reduced or increased AOR for fatigue were employment status, education status, type of MS, DMD use, smoking status, alcohol use, physical activity, vitamin D supplementation, meditation, social support, BMI, and diet (Table 4). The main variables associated with reduced or increased AOR for depression were age, employment status, years since diagnosis, DMD use, smoking status, alcohol use, physical activity, social support, and diet (Table 4). The main variables associated with reduced or increased AOR for unassisted walking ability were region, gender, education status, years since diagnosis, DMD use, smoking status, alcohol consumption, physical activity level, and BMI (Table 4).

### Modifiable Variables Associated with Health Outcomes

Current smoking was associated with a 7–8 point lower score on both QOL domains, and 2.2 times higher odds for depression risk compared to never smoking. Moderate/high alcohol use was associated with 3 points higher physical QOL score, 2 points higher mental health QOL score, 24% lower odds of disability, 38% lower odds of fatigue, and 29% lower odds of depression compared to low alcohol use. It must be noted that very few people consumed high levels of alcohol.

High levels of physical activity were associated with 15 points higher physical QOL score, 7 points higher mental health QOL score, 72% lower odds of disability, 71% lower odds of clinically significant fatigue, and 60% lower odds of depression risk compared to low levels of physical activity. Moderate levels of physical activity were also associated with higher physical and mental health QOL scores, and lower odds of disability, fatigue, and depression. Vitamin D supplementation of 1–5,000 IU was associated with 30% lower odds of fatigue and vitamin D supplementation of >5,000 IU was associated with 38% lower odds of fatigue compared to no vitamin D supplementation. Having two or more people to rely on for support was associated with 4 points higher physical QOL score, 7 points higher mental health QOL score, and 55% lower odds of depression compared to having zero or no support people.

Overweight BMI was associated with 38% higher odds of fatigue compared to normal BMI, and obese BMI associated with 93% higher odds of fatigue and 45% higher odds of disability compared to normal BMI. Overweight and obese BMI were associated with 3 and 6 points lower physical QOL scores, respectively, and 3 and 5 points lower mental health QOL scores, respectively, compared to normal BMI. Healthier dietary habits scores were associated with 19% lower odds of fatigue and 16% lower odds of depression.

Disease-modifying drug use for more than 12 months was associated with 48% higher odds of clinically significant fatigue and 41% higher odds of depression compared to no DMD use. However, DMD use for more than 12 months was associated with 29% lower odds of disability compared to no DMD use.

## Discussion

A previous study from our group identifying comorbidities in PwMS using HOLISM data reported international regional differences in the comorbidities assessed and in BMI (14). The question of whether there were international regional differences in disability, QOL, fatigue, and depression in PwMS, and what their determinants might be arose from this study.

Disability (inability to walk unassisted), depression, and clinically significant fatigue were most common in North American, followed by European and Australasian participants of HOLISM. Small but statistically significant differences were seen in physical and mental QOL in a similar pattern. A wide range of prevalence is reported for depression, comorbidities, and other health outcomes in different countries (27), and QOL differences between countries have also been reported (28). These may be attributable to different study inclusion criteria, recruitment methods but may also reflect population differences in demographics, disease characteristics, lifestyles, or other factors. The international breadth of the HOLISM study enabled these questions to be studied in detail in this article.

Multivariable regression analyses showed that QOL, fatigue, and depression were associated with sociodemographics, disease characteristics, and modifiable lifestyle factors. The sociodemographic and disease variables associated with better health outcomes were younger age, female gender, current employment, higher level of education, shorter disease duration, and non-progressive type of MS. DMD use was associated with higher odds of being fatigued or depressed, which may be common side effects of DMDs (29, 30), but lower odds for inability to walk unassisted. Lifestyle factors, of special interest given their modifiable character, associated with better health outcomes were non-smoking status, moderate alcohol use (vs. low), high level of physical activity (vs. low), normal BMI, healthier diet, vitamin D supplementation, and having several support people, in line with current literature (1, 2, 7). Regular meditation was associated with increased odds for fatigue, which may be due to reverse causality as our analysis here is a cross-sectional; a trial is currently underway which may provide further insights (31).

However, even after adjusting for these factors, disability differences between those in North America, Europe, and Australasia still existed. There may be determinants other than the variables included in our models that could explain these significant geographical differences in disability in our sample. It could be speculated that health system factors and accessibility, economic status, access to MS support facilities, previous medication use, and local culture may influence disability in PwMS and may contribute to the differing levels of disability between regions. Incidence of a number of infections also differs between countries and might also be associated with regional differences in disability (32). Future studies should focus on examining these and other specific factors that may be implicated in the observed differences.

A consistent approach to measurement, conducted in large samples, ideally registries, would yield more valuable results (27). Understudied regions such as Asia and Africa still warrant further study, although it is likely that there are fewer studies in these areas because they are less developed or closer to the equator, where the prevalence of MS is lower (4).

The findings of this study have significant clinical implications. From a clinical perspective, physicians should be aware that differences in QOL, depression, and fatigue in PwMS are associated with sociodemographic and disease variables, as well as differences in lifestyle factors. Modification of these lifestyle factors, particularly smoking cessation, regular physical activity, healthy diet, and normal weight, may contribute to improved QOL and reductions in depression and fatigue. While we have previously shown important associations between lifestyle and health outcomes in our sample (11, 13, 25, 26, 33), there are likely other factors at play in explaining differences in disability between regions including variation in health services and access to health care and medications, cultural factors, infection prevalence, and chronic disease prevalence. These factors should be the focus of future research and may provide alternative strategies as part of a comprehensive approach to management of patients with MS.

### Limitations

Participants who were not from the three main regions were excluded from this study. Thus, results are not generalizable to PwMS living in other countries. Most PwMS live in the three regions studied, which are generally developed countries and further from the equator. Data provided via the online survey were self-reported and therefore were unable to be verified. Where possible validated tools were used, but for some domains that have not been extensively investigated such as vitamin D supplementation and meditation, validated tools were not available. We dichotomized PDDS responses for ease of analysis, however, this has not previously been validated. Despite the varied backgrounds of participants and large sample, our findings may not be generalizable to all PwMS, as respondents were all English-speaking, mostly young women, overall highly educated, and able to complete the survey online. Selection bias is therefore a likely limitation of the study. Ethnicity was not taken into account and may be a variable of interest for future research. Causality cannot be inferred between factors assessed due to this cross-sectional study design. Temporality is likely to be a significant limitation particularly in cross-sectional analysis of disability due to the time taken to develop disability. Longitudinal data from HOLISM may clarify the associations found in this study and the issue of temporality. Our analyses here were restricted to complete case analyses, that is, the analyses were restricted to participants with complete data, however, the Table S1 in Supplementary Material shows that the sociodemographics and disease characteristics of the participants with and without any missing values are not systematically different.

## Conclusion

We observed differences in physical and mental health-related QOL, disability, clinically significant fatigue, and depression risk between three regions in an international sample of PwMS. However, after adjusting for sociodemographic characteristics, disease profile, and modifiable lifestyle factors, these differences disappeared for all health outcomes except disability. Unlike sociodemographics and stable disease characteristics, lifestyle factors can be modified. These results support existing evidence that smoking, low physical activity, and normal weight are associated with better health outcomes. Differences in disability may be associated with other unmeasured factors, such as differences in health system factors and accessibility, economic variations, access to support facilities, medication, and local culture between countries. Future studies are needed to investigate these differences and potentially identify other modifiable factors related to disability.

## Ethics Statement

This study was carried out in accordance with the recommendations of Health Sciences Human Ethics Sub-Committee at the University of Melbourne with digitally indicated informed consent from all subjects. All subjects gave written informed consent in accordance with the Declaration of Helsinki. The protocol was approved by the Health Sciences Human Ethics Sub-Committee at the University of Melbourne (Ethics ID: 1545102).

## Author Contributions

GJ conceived and obtained funding for the study. GR, AM, GJ, CM, and TW contributed to the design of the study. GR and AM were involved in data collection, analysis of data, and drafted the manuscript with PJ and CM. AL contributed to the data analysis. All the authors edited and approved the final manuscript.

## Conflict of Interest Statement

GJ receives royalties for his books Overcoming Multiple Sclerosis and Recovering from Multiple Sclerosis. GJ, SN, and KT have received remuneration for conducting lifestyle educational interventions for people with MS. The other authors declare no competing interests.

## Abbreviations

BMI, body mass index; CI, confidence interval; DHQ, diet habits questionnaire; DMDs, disease-modifying drugs; EDSS, expanded disability status scale; FSS, Fatigue Severity Scale; HOLISM, health outcomes and lifestyle in a sample of people with multiple sclerosis; IPAQ, International Physical Activity Questionnaire; MS, multiple sclerosis; OR, odds ratio; PDDS, Patient Determined Disease Step; PHQ-2, Patient Health Questionnaire-2; PwMS, people with multiple sclerosis; QOL, quality of life; SIMSS, Single Item Measure of Social Support; US, United States.
