# Supplementary material for: International Differences in Multiple Sclerosis Health Outcomes and Associated Factors in a Cross-sectional Survey
Source: Front Neurol. 2017 May 31;8:229. doi: 10.3389/fneur.2017.00229 (PMC5450014; doi:10.3389/fneur.2017.00229)
Supplement: Supplementary file 1 [file Table_1.DOCX]

Supplementary Table 1. Summary statistics of demographics and disease characteristic for missing and non-missing data in Disability regression model

| **Variable** | **Missing N (%) or mean (95% CI)** N=541 | **Non-missing N (%) or mean (95% CI)** N=1868 |
| --- | --- | --- |
|  | Total | Total |
| Years of Age | 45.7 (44.8-46.7) | 45.7 (45.2-46.1) |
| Female | 305 (80.7%) | 1550 (83.0%) |
| Years since diagnosis |  |  |
| Less than 3 years | 138 (28.0%) | 550 (29.4%) |
| 4-6 years | 104 (21.1%) | 422 (22.6%) |
| 7-12 years | 111 (22.5%) | 465 (24.9%) |
| 13-54 years | 140 (28.4%) | 431 (23.1%) |
| Education |  |  |
| No school/Primary/Secondary | 133 (25.2%) | 459 (24.6%) |
| Vocational training | 87 (16.5%) | 299 (16.0%) |
| Bachelor degree | 203 (38.5%) | 662 (35.4%) |
| Postgraduate | 104 (19.7%) | 448 (24.0%) |
| Current DMD use |  |  |
| None | 171 (55.0%) | 942 (50.4%) |
| Less than 12 months | 38 (12.2%) | 290 (15.5%) |
| More than 12 months | 102 (32.8%) | 636 (34.1%) |

Supplementary Table 2. Summary statistics of demographics and disease characteristics for missing and non-missing data in Fatigue regression model

| **Variable** | **Missing N (%) or mean (95% CI)** N=670 | **Non-missing N (%) or mean (95% CI)** N=1739 |
| --- | --- | --- |
| Years of Age | 45.9 (45.1-46.8) | 45.6 (45.1-46.1) |
| Female | 415 (81.9%) | 1440 (82.8%) |
| Years since diagnosis |  |  |
| Less than 3 years | 172 (27.7%) | 516 (29.7%) |
| 4-6 years | 135 (21.7%) | 391 (22.5%) |
| 7-12 years | 146 (23.5%) | 430 (24.7%) |
| 13-54 years | 169 (27.2%) | 402 (23.1%) |
| Education |  |  |
| No school/Primary/Secondary | 169 (25.8%) | 423 (24.3%) |
| Vocational training | 110 (16.8%) | 276 (15.9%) |
| Bachelor degree | 250 (38.1%) | 615 (35.4%) |
| Postgraduate | 127 (19.4%) | 425 (24.4%) |
| Current DMD use |  |  |
| None | 246 (55.9%) | 867 (49.9%) |
| Less than 12 months | 57 (13.0%) | 271 (15.6%) |
| More than 12 months | 137 (31.1%) | 601 (34.6%) |
| Employment status |  |  |
| Full time/Part time | 308 (50.8%) | 978 (56.2%) |
| Student/Stay at home carer | 54 (8.9%) | 185 (10.6%) |
| Unemployed | 64 (10.6%) | 123 (7.1%) |
| Retired due to age/disability | 180 (29.7%) | 453 (26.1%) |
| Type of MS |  |  |
| Benign | 25 (4.0%) | 69 (4.0%) |
| Relapsing/Remitting | 379 (60.7%) | 1082 (62.2%) |
| Primary Progressive | 43 (6.9%) | 130 (7.5%) |
| Secondary Progressive | 75 (12.0%) | 195 (11.2%) |
| Progressive | 14 (2.2%) | 32 (1.8%) |
| Unsure/other | 88 (14.1%) | 231 (13.3%) |

Supplementary Table 3. Summary statistics of demographics and disease characteristics for missing and non-missing data in Depression regression model

| **Variable** | **Missing N (%) or mean (95% CI)** N=626 | **Non-missing N (%) or mean (95% CI)** N=1783 |
| --- | --- | --- |
|  | Total | Total |
| Years of Age | 45.7 (44.8-46.6) | 45.7 (45.2-46.2) |
| Female | 373 (80.6%) | 1482 (83.1%) |
| Years since diagnosis |  |  |
| Less than 3 years | 164 (28.4%) | 524 (29.4%) |
| 4-6 years | 125 (21.6%) | 401 (22.5%) |
| 7-12 years | 131 (22.7%) | 445 (25.0%) |
| 13-54 years | 158 (27.3%) | 413 (23.2%) |
| Education |  |  |
| No school/Primary/Secondary | 162 (26.5%) | 430 (24.1%) |
| Vocational training | 99 (16.2%) | 287 (16.1%) |
| Bachelor degree | 232 (37.9%) | 633 (35.5%) |
| Postgraduate | 119 (19.4%) | 433 (24.3%) |
| Current DMD use |  |  |
| None | 218 (55.1%) | 895 (50.2%) |
| Less than 12 months | 53 (13.4%) | 275 (15.4%) |
| More than 12 months | 125 (31.6%) | 613 (34.4%) |
| Employment status |  |  |
| Full time/Part time | 284 (50.5%) | 1002 (56.2%) |
| Student/Stay at home carer | 50 (8.9%) | 189 (10.6%) |
| Unemployed | 63 (11.2%) | 124 (7.0%) |
| Retired due to age/disability | 165 (29.4%) | 468 (26.3%) |
| Type of MS |  |  |
| Benign | 24 (4.1%) | 70 (3.9%) |
| Relapsing/Remitting | 347 (59.8%) | 1114 (62.5%) |
| Primary Progressive | 43 (7.4%) | 130 (7.3%) |
| Secondary Progressive | 68 (11.7%) | 202 (11.3%) |
| Progressive | 13 (2.2%) | 33 (1.9%) |
| Unsure/other | 85 (14.7%) | 234 (13.1%) |
| Marital Status |  |  |
| Married/partnered/Cohabiting | 382 (64.9%) | 1372 (77.0%) |
| Single | 118 (20.0%) | 222 (12.5%) |
| Separated/divorced/widower | 89 (15.1%) | 189 (10.6%) |

Supplementary Table 4. Summary statistics of demographics and disease characteristics for missing and non-missing data in Physical Health QOL regression model

| **Variable** | **Missing N (%) or mean (95% CI)** N=803 | **Non-missing N (%) or mean (95% CI)** N=1606 |
| --- | --- | --- |
|  | Total | Total |
| Years of Age | 47.0 (46.2-47.8) | 45.1 (44.6-45.6) |
| Female | 541 (84.5%) | 1314 (81.8%) |
| Years since diagnosis |  |  |
| Less than 3 years | 199 (26.4%) | 489 (30.5%) |
| 4-6 years | 170 (22.5%) | 356 (22.2%) |
| 7-12 years | 175 (23.2%) | 401 (25.0%) |
| 13-54 years | 211 (28.0%) | 360 (22.4%) |
| Education |  |  |
| No school/Primary/Secondary | 211 (26.7%) | 381 (23.7%) |
| Vocational training | 127 (16.1%) | 259 (16.1%) |
| Bachelor degree | 294 (37.3%) | 571 (35.6%) |
| Postgraduate | 157 (19.9%) | 395 (24.6%) |
| Current DMD use |  |  |
| None | 327 (57.1%) | 786 (48.9%) |
| Less than 12 months | 73 (12.7%) | 255 (15.9%) |
| More than 12 months | 173 (30.2%) | 565 (35.2%) |
| Employment status |  |  |
| Full time/Part time | 370 (50.1%) | 916 (57.0%) |
| Student/Stay at home carer | 62 (8.4%) | 177 (11.0%) |
| Unemployed | 80 (10.8%) | 107 (6.7%) |
| Retired due to age/disability | 227 (30.7%) | 406 (25.3%) |

Supplementary Table 5. Summary statistics of demographics and disease characteristics for missing and non-missing data in Mental Health QOL regression model

| **Variable** | **Missing N (%) or mean (95% CI)** N=638 | **Non-missing N (%) or mean (95% CI)** N=1771 |
| --- | --- | --- |
|  | Total | Total |
| Years of Age | 46.0 (45.1-46.8) | 45.6 (45.1-46.1) |
| Female | 385 (80.9%) | 1470 (83.1%) |
| Years since diagnosis |  |  |
| Less than 3 years | 164 (27.8%) | 524 (29.6%) |
| 4-6 years | 130 (22.0%) | 396 (22.4%) |
| 7-12 years | 133 (22.5%) | 443 (25.0%) |
| 13-54 years | 164 (27.8%) | 407 (23.0%) |
| Education |  |  |
| No school/Primary/Secondary | 163 (26.1%) | 429 (24.2%) |
| Vocational training | 105 (16.8%) | 281 (15.9%) |
| Bachelor degree | 237 (37.9%) | 628 (35.5%) |
| Postgraduate | 120 (19.2%) | 432 (24.4%) |
| Current DMD use |  |  |
| None | 227 (55.5%) | 886 (50.1%) |
| Less than 12 months | 54 (13.2%) | 274 (15.5%) |
| More than 12 months | 128 (31.3%) | 610 (34.5%) |
| Employment status |  |  |
| Full time/Part time | 292 (50.8%) | 994 (56.2%) |
| Student/Stay at home carer | 50 (8.7%) | 189 (10.7%) |
| Unemployed | 64 (11.1%) | 123 (7.0%) |
| Retired due to age/disability | 169 (29.4%) | 464 (26.2%) |
| Type of MS |  |  |
| Benign | 26 (4.4%) | 68 (3.8%) |
| Relapsing/Remitting | 349 (58.9%) | 1112 (62.8%) |
| Primary Progressive | 43 (7.3%) | 130 (7.3%) |
| Secondary Progressive | 75 (12.7%) | 195 (11.0%) |
| Progressive | 13 (2.2%) | 33 (1.9%) |
| Unsure/other | 87 (14.7%) | 232 (13.1%) |
| Marital Status |  |  |
| Married/partnered/Cohabiting | 393 (65.3%) | 1361 (76.9%) |
| Single | 118 (19.6%) | 222 (12.5%) |
| Separated/divorced/widower | 91 (15.1%) | 187 (10.6%) |
